# Eating behaviour disorders among adolescents in a middle school in Dongfanghong, China

**DOI:** 10.1186/s40337-017-0175-x

**Published:** 2017-10-26

**Authors:** Tingting Feng, Dawit Shawel Abebe

**Affiliations:** 1 University of Oslo, Oslo, Norway; 2 Norwegian Social Research (NOVA), Oslo and Akershus University College of Applied Sciences, Oslo, Norway

**Keywords:** Disordered eating behaviours, Associated factors, Adolescents, China

## Abstract

**Background:**

Disordered eating behaviours (DEB) are significant public health challenges among adolescents. DEB are prevalent among Chinese young people and replication epidemiological studies in DEB are needed due to ongoing rapid modernization and socio-economic change. In addition, there has been no prevention or intervention programs for DEB in most of rural areas in China and research in DEB in rural China is limited. More research in DEB in rural China is needed for increased awareness of prevention strategies. The objectives of the study are to examine the prevalence and associated factors of DEB among rural Chinese adolescents.

**Methods:**

Four hundred and sixty-six students aged 11–18 years old from a middle school in rural China were invited to complete a self-reported questionnaire that included measures on DEB and psychosocial factors. The SCOFF questionnaire was applied to measure DEB (i.e., a score of > = 2 indicates a likely case of DEB). Logistic regression models were applied for data analyses. A *p*-value <0.05 was regarded as statistically significant.

**Results:**

There were 389 adolescents (11–18 years) included in this study with the prevalence of DEB being 28.8%. No significant differences were found between male adolescents (30.5%) and female adolescents (27.1%). Independently, perceived overweight (OR = 2.80, 95% CI = 1.05–7.48), negative affect (OR = 1.07, 95% CI = 1.03–1.12), body dissatisfaction (OR = 0.96, 95% CI = 0.93–0.99), and watching TV (OR = 2.40, 95% CI = 1.11–5.18) were significantly (*p* < 0.05) associated with DEB.

**Conclusion:**

This study indicates a high prevalence rate of DEB among rural adolescents and associated factors of DEB from a school-based sample. Such findings imply that local public health systems should pay more attention to DEB and design prevention and intervention strategies for DEB.

**Electronic supplementary material:**

The online version of this article (10.1186/s40337-017-0175-x) contains supplementary material, which is available to authorized users.

## Plain English summary

Disordered Eating Behaviours (DEB) start to appear during adolescence and bring serious consequences. Previous studies showed DEB is becoming common in China, and even more prevalent than that is in Western countries. However, limited research has been conducted among adolescents in rural China. The current study aimed to establish the prevalence of DEB and associated psychosocial factors among rural adolescents. Results from the current research showed a high prevalence rate of DEB among rural adolescents and associated psychosocial factors of DEB.

## Background

Disordered Eating Behaviours (DEB) are abnormal eating attitudes and behaviours which have much in common with diagnosed eating disorders [1].These can have serious implications which relate to depression, substance abuse, suicidal behaviours, social isolation and which may develop into an eating disorder [2, 3].

DEB has long been regarded as strongly related to Western cultures but according to a review in the most recent published epidemiologic data relevant to eating disorders in Asia and the Pacific, abnormal eating behaviours found to be highly prevalent in China [4]. Traditional Chinese culture considers plumpness as beautiful and slimness as reflecting poverty. However, China has been dramatically influenced by Western cultures for the past 39 years due to the ‘open-door policy’. The thin-ideal has come to overshadow standards for beauty which contributes to body dissatisfaction and DEB among females [5]. Accordingly, a number of Chinese studies report a high prevalence of DEB [6–13]. Specifically, female adolescents living in Hong Kong have been found to have the highest prevalence of DEB (10.8%), followed by those from Shenzhen (5.2%) and from Hunan (2.5%) [6]. Two school-based studies in Taiwan revealed DEB in 17.1% and 10.4% of high school female adolescents [7, 8]. Further, a school-based study in Hong Kong revealed that DEB were present in 3.9% of adolescent males and 6.5% of adolescent females [9]. Three school-based studies in Mainland China have also reported that the prevalence of DEB varied from 2.3% to 17% among middle school and college students [10–12]. Western studies have revealed DEB in 29.4% of female adolescents, specifically 14.9% of male adolescents in Germany, and 56% of female adolescents and 28% of male adolescents in US [14, 15]. However, few studies in DEB have included male adolescents in China [9, 13]. The variations in measuring instrument, sample characteristics, methodological approaches and study areas, however, may limit the comparability of prevalence across studies. The updated epidemiologic review in 2016 suggested that fat concern and weight control behaviours are prevalent among Chinese young people and replication epidemiological studies in eating disturbances are needed due to ongoing rapid modernization and socio-economic change [4].

Psychosocial risk factors are strongly associated with a high prevalence of DEB among adolescents [13, 16]. For instance, longitudinal studies conducted in Southern China found body dissatisfaction, negative affect, perceived sociocultural pressures, and high socioeconomic status (SES) all have significant associations with DEB. [13, 17].

A comparative study among female adolescents in three different socioeconomically communities of China documented that those in rural areas had fewer concerns about body fat and demonstrated a lower prevalence rate of DEB than those in developed cities [6]. Socioeconomic development in China is growing very fast nowadays and the rapid ongoing socioeconomic transition has influenced rural China to a large extent. Rural populations now have more opportunities to access the mass media, which may relate to a higher risk of DEB. T Jackson and H Chen have reported that Chinese middle adolescent girls reported higher levels of appearance experiences related to mass media and DEB [18], and pressure from mass media related to DEB have been observed in Chinese adolescent boys [19]. There are some studies examining the epidemiology of eating disorders in rural China [17, 20, 21], but little research has focused on epidemiology of DEB in rural China. In more than 20 years, there has been only one study which has focused on DEB among Chinese female adolescents in a rural area [6]. Further, given that little attention has been paid to DEB in both genders in rural China, it is necessary to re-investigate the situation under current influences. The present study, therefore, aimed to examine prevalence and associated psychosocial factors of DEB among male adolescents and female adolescents at a Chinese middle school in a rural area. This may contribute to the updated information in the epidemiology of DEB in China and also respond to the updated review’s request for replication studies, that is this review suggested further replication epidemiological studies in eating disturbances due to ongoing rapid modernization and socio-economic change [4].

## Methods

### Participants and study procedure

All students from a middle school – a secondary high school – in Dongfanghong town (a less economically developed rural area), Heilongjiang province were invited to participate in the study. In 2015, the per capita annual income in our sample area was 30,977 RMB (or $4509). The school that was studied was the only middle school in this area. Four hundred and sixty-six students aged 11–18 years old were invited to complete an anonymous, self-reported questionnaire covering a wide range of topics including DEB and related psychosocial behaviours on a voluntary basis.

Before starting the research, schoolteachers were trained by researchers and schoolteachers explained the study to students and were able to answer students’ questions. During the study, the confidentiality and the anonymity of participants were assured. With the assistance of schoolteachers, the questionnaires were administered in September 2015 – at the beginning of autumn semester. The class teachers decided the time when questionnaires were conducted. They usually selected self-learning class (students studied by themselves) and the time of self-learning class was different among class-rooms. The students used around 1 h -2 h to finish the questionnaires. The class teachers collected the questionnaires following completion and returned them to the researcher. Of 466 questionnaires that were issued, 389 valid questionnaires were returned (a response rate of 83.5%).

### Outcome variables

SCOFF: The SCOFF questionnaire is an effective tool that can screen DEB quickly [22]. It includes five items addressing the main features of DEB [22]. See the Additional file 1: Table S1 for the descriptive statistics of each item.

One point for every “yes”; a score of > = 2 indicates a likely case of DEB. The scale has been validated and has demonstrated effective specificity in identifying DEB [23]. The Chinese version of the SCOFF questionnaire has been proved to be effective for detecting potential DEB [24].

### Independent variables

Body Mass Index (BMI) was computed from self-reported measures of height and weight. Self-reported BMI has been demonstrated to be a reliable measure of BMI [25]. The classification of BMI was made according to BMI cut-offs by the Working Group on Obesity in China (WGOC) [26]: ≤ 18.49 kg/m^2^ (underweight), 18.50–23.99 kg/m^2^ (normal weight), 24–27.99 (overweight), ≥28 (obesity). We combined the overweight group and the obesity group into one category due to the small number size in the obesity group.

Participants were asked to describe their weight perceptions. Responses were ranged into three groups: underweight, average /normal, and overweight.

Compared weight perceptions with actual BMI, weight misconception was ranged into four groups: misconception of underweight, overweight, normal weight and correct weight conception. See the Additional file 2: Table S2 for the association between BMI and perceived weight categories.

Body dissatisfaction was examined using a Body Part Satisfaction Scale with each response from 1 = extremely dissatisfied to 6 = extremely satisfied [27]. The scale consists of eight items rating the individual’s level of satisfaction with the following body areas: face, upper torso, middle torso, lower torso, muscle tone, weight, height and overall appearance. A high score indicates a higher level of body satisfaction. The scale has shown reliable psychometrics [27]. The Chinese version of this scale has been tested and has strong reliability [18]. The internal consistency of the scale was 0.90.

Depressive symptoms were measured by the Positive and Negative Affect Schedule [28]. This schedule is a 20-item test with 10 items for negative affect and positive affect, respectively. Participants were asked to describe the extent to which they had varying feelings and emotions in the past month from 1 = very slightly or not at all to 5 = extremely. The scale has shown good validity and acceptable psychometric properties [28]. The original scale has been tested on Chinese samples [19].

The Perceived Sociocultural Pressure Scale was used to assess pressures to be thin from family, friends, dating partners and media [18]. The questionnaire has 10 items that investigate perceived sociocultural pressures to be thin. An example of an item is: “I’ve noticed a strong message from my family to have a thin body”. Three items are for family and friends, respectively. Two items are for dating partners and media, respectively. Responses ranged from 1 = not at all to 5 = extremely. Higher scores indicate more perceived pressure. Internal consistency (α = 0.83), test-retest reliability (*r* = 0.93) and predictive validity of this scale have been documented [29]. The scale has been tested and used in Chinese samples [19].

Media use was assessed by a media exposure questionnaire. Participants were asked: whether they watch television and DVDs, whether they use the Internet, whether they use chat rooms and play computer games, as well as how long they spent on media. The questionnaire was developed from a Chinese study [5].

Parental socioeconomic status (SES) was determined on the adolescents’ report of parents’ occupation. Occupations were categorized according to the classification by the Chinese National Bureau of Statistics [30] with nine items to be selected. Four groups were created based on the salary of occupations: professional, service, labourers, and unemployed. The variables of mother’s job and father’s job were merged into one variable (parents’ occupation), recording the higher of the two values.

Age and sex were also recorded.

### Statistical analysis

Chi-square analysis was used to compare differences in prevalence of DEB and for comparing perceived weight categories to BMI categories. To examine associated factors of DEB, first, a univariate logistic regression was employed to examine the association between each of the independent variable and DEB, and then multiple logistic analysis was performed. A forward stepwise multiple logistic regression was performed to determine factors significantly associated with DEB, including independent variables based on univariate logistic regression results (cut-off point *p* ⩽ 0.25). Age and gender were controlled. The final model was built based on this forward stepwise multiple logistic regression (*p* ⩽ 0.05). The multi-collinearity among associated factors was examined with *collin* command in Stata (mean variance inflation factor (VIF) = 1.43, correlation matrix = 0.0528). Secondly, Model fit to the data (validity and reliability) was assessed by the likelihood ratio χ^2^ test, Hosmer-Lemeshowgoodness-of-fit test and receiver operating characteristics (ROC) curve. *P* ⩽ 0.05 was regarded as statistically significant and odds ratio (OR) with 95% confidence intervals (95% CI) were calculated. Data analyses were conducted with the Statistical Package for the Social Sciences (SPSS) 22.0 and StataSE 14.

### Ethical clearance

The study was approved by the Regional Committee for Medical Research Ethics in Norway, and the Committee for the middle school in Dongfanghong, China.

## Results

### Descriptive summary of the study participants

Table 1 presents the descriptive summary of study participants (*N* = 389).

**Table 1:** Descriptive summary of the study participants (*N* = 389)

Variables	N	Percent	M	sd
Female	190	48.8		
SCOFF total score (five tiems)			1.0	1.1
0	172	44.2		
1	105	27.0		
2	67	17.2		
3	32	8.2		
4	12	3.1		
5	1	0.3		
Age(year)			15.1	1.7
Early Adolescence [11–13]	95	24.4		
Middle Adolescence [14–16]	198	50.9		
Late Adolescence [17, 18]	96	24.7		
BMI(kg/m^2^)			20.5	4.7
Underweight	140	36.0		
Normal Weight	169	43.4		
Overweight	77	19.8		
Perceived Weight				
Underweight	61	15.7		
Normal Weight	214	55.0		
Overweight	114	29.3		
Weight Misconception				
Misconception of Underweight	18	4.6		
Misconception of Normal Weight	102	26.2		
Misconception of Overweight	57	14.7		
Correct Weight Conception	207	53.2		
Parents’ Occupation				
Professional	115	29.6		
Service	72	18.5		
Labourers	48	12.3		
Unemployed	154	39.6		
With DEB	112	28.8		
Pressure				
Family			1.9	1.1
Friend			1.8	1.0
Dating			1.5	1.0
Media			1.6	1.1
Watch TV	323	83.0		
Watch DVD	74	19.0		
Surfing Online	353	90.7		
Chat Online	316	81.2		
Play Online	253	65.0		
Hours on Media average - 0-4.9 h	315	81		
Hours on Media Total			14.1	9.1
Body Satisfaction			31.1	8.4
Face			4.0	1.3
Upper torso			4.0	1.3
Middle torso			4.0	1.4
Lower torso			4.0	1.4
Muscle tone			3.7	1.4
Height			3.8	1.4
Weight			3.7	1.5
Overall appearance			4.1	1.2
Positive Affect			29.6	7.5
Negative Affect			21.2	7.2

*N* number, *M* mean, *sd* standard deviation

*Pressure* perceived sociocultural pressure to thin body and weight loss

*Hours on Media Total* time on Internet working days + time on Internet weekends + time on TV working days + time on TV weekends

*Hours on Media average* Hours on Media Total ÷ 4

### Prevalence of disordered eating behaviours

Table 2 presents the prevalence of DEB according to the participants’ general information by using chi-square analysis. The prevalence of DEB among adolescents was 28.8%. The prevalence of DEB among male adolescents and female adolescents was 27.1% and 30.5%, respectively; no significant differences were found based on gender. No significant differences of prevalence of DEB were found across different age groups. Significant differences in the prevalence of DEB were found in different BMI groups, χ^2^ (2) = 14.896, *p* = .001. Overweight adolescents (42.9%) had a significantly higher prevalence of DEB as compared to underweight adolescents (18.6%). Significant differences in the prevalence of DEB were also found in different perceived weight groups, χ^2^ (2) = 51.524, *p* < .001. Adolescents in the perceived overweight group (54.4%) had a significantly higher prevalence of DEB than those among the perceived underweight group (18.0%). Significant differences in the prevalence of DEB were found in different weight misconception groups, χ^2^ (2) = 43.399, *p* < .001. Adolescents with overweight misconception (59.4%) had a significantly higher prevalence of DEB than those without overweight misconception. There were no significant differences in the prevalence of DEB among students across different SES groups. Associations between misconception of over−/under-weight and DEB by gender is shown in the Additional file 3: Table S3.

**Table 2:** Prevalence of disordered eating behaviours across participants’ general information

	With DEB	Without DEB	Chi-square
	% (N)	% (N)	Statistic
Gender			0.545
Female	30.5 (58)	69.5 (132)	
Male	27.1 (54)	72.9 (145)	
Age			4.919
Early Adolescent [11–13]	24.2 (23)	75.8 (72)	
Middle Adolescent [14–16]	26.8 (53)	73.2 (145)	
Late Adolescent [17, 18]	37.5 (36)	62.5 (60)	
BMI			14.896**
Underweight	18.6 (26)	81.4 (114)	
Normal Weight	30.8 (52)	69.2 (117)	
Overweight	42.9 (33)	57.1 (44)	
Perceived Weight			51.524***
Underweight	18.0 (11)	82.0 (50)	
Normal Weight	18.2 (39)	81.8 (175)	
Overweight	54.4 (62)	45.6 (52)	
Weight Misconception			41.521***
Misconception of Underweight	22.2 (4)	77.8 (14)	
Misconception of Normal Weight	13.7 (14)	86.3 (88)	
Misconception of Overweight	61.4 (35)	38.6 (22)	
Correct Weight Conception	27.5 (57)	72.5 (150)	
Parents’ Occupation			2.218
Professional	33.9 (39)	66.1 (76)	
Service	25.0 (18)	75.0 (54)	
Labourers	27.1 (13)	72.9 (35)	
Unemployed	27.3 (42)	72.7 (112)	

*N* number of participants

*p < 0.05, **p < 0.01, ***p < 0.001

### Psychosocial factors independently predicting disordered eating behaviours

Table 3 presents results from univariate and multiple logistic regression analyses reporting psychosocial associated factors of DEB. The final multiple regression model included perceived weight, body dissatisfaction, negative affect and watching TV. The risk of having DEB among the perceived overweight group was 2.8 times higher than among those in the perceived underweight group. Students with higher levels of body dissatisfaction were significantly more likely to develop DEB compared to those with lower levels of dissatisfaction. Students with a higher negative affect were significantly more likely to develop DEB compared with those with a lower negative affect. Students watching TV were almost three times more likely to report DEB than students who did not watch TV. The corresponding coefficients and standard errors in the multiple logistic regression model are shown in the Additional file 4: Table S4.

**Table 3:** Multiple logistic regression model showing factors associated with disordered eating behaviours

	Univariate Logistic Regression Analysis per Independent Variable		Forward Multivariate Stepwise Logistic Regression Analysis	
	OR (95% CI)	*P*	OR (95% CI)	*P*
BMI				
Underweight	1		–	–
Normal Weight	1.95 (1.14–3.33)	.015**	–	–
Overweight	3.29 (1.77–6.11)	<.001****	–	–
Perceived Weight				
Underweight	1		1	
Normal Weight	1.01 (0.48–2.12)	.973	0.79 (0.34–1.86)	.589
Overweight	5.42 (2.56–11.47)	<.001****	2.80 (1.05–7.48)	.041**
Weight Misconception				
Correct Weight Conception	1		–	–
Misconception of Underweight	0.75 (0.24–2.38)	.628	–	–
Misconception of Normal Weight	0.42 (0.22–0.80)	.008***	–	–
Misconception of Overweight	4.19 (2.27–7.74)	<.001****	–	–
Pressure from family	2.17 (1.75–2.70)	<.001****	–	–
Pressure from friends	2.48 (1.93–3.18)	<.001****	–	–
Pressure from dating	1.86 (1.48–2.34)	<.001****	–	–
Pressure from media	1.90 (1.53–2.36)	<.001****	–	–
Watch TV				
No	1		1	
Yes	1.80 (0.94–3.46)	.076*	2.40 (1.11–5.18)	.026**
Watch DVD				
No	1		–	–
Yes	1.67 (0.98–2.85)	.058*	–	–
Chat Online				
No	1		–	–
Yes	2.35 (1.21–4.57)	.011**	–	–
Play Online				
No	1		–	–
Yes	1.34 (0.84–2.14)	.23*	–	–
Body Satisfaction	0.92 (0.89–0.95)	<.001****	0.96 (0.93–0.99)	.019**
Positive Affect	0.96 (0.93–0.99)	.005***	–	–
Negative Affect	1.10 (1.06–1.14)	<.001****	1.07 (1.03–1.12)	<.001****

*OR* odds ratio, *CI* confidence interval

*Pressure* perceived sociocultural pressure to thin body and weight loss

*p ⩽ 0.25, **p < 0.05, ***p < 0.01, ****p < 0.001

The reliability and the validity of the multiple logistic regression model was assessed by employing post-estimation statistics (likelihood ratio χ^2^ test significant, χ^2^ = 107.925, degrees of freedom (df) = 9, *p* < 0.001; Hosmer-Lemeshow χ^2^ test = 10.196, df = 8, *p* = 0.252). The Hosmer-Lemeshow test of the goodness-of-fit suggests the model is a good fit to the data (small Chi-squared values with larger *p*-value closer to 1). Area under the ROC curve (AUC) was 0.825 with 95% confidence interval (0.781, 0.869), which is a prediction power for the model.

## Discussion

The main findings of this school-based study reveal a high prevalence rate of DEB among male adolescents (27.1%) and female adolescents (30.5%). The study also found that perceived weight, negative affect, body dissatisfaction, and watching TV were significantly associated with DEB among adolescents.

The prevalence estimates of the current study are among a wide range of previous school-based studies conducted in China (2.26%–58.3%) [10, 31–33]. The available information on DEB in rural China has been reported by Sing Lee and Antoinette M. Lee [6]. In their study, 266 school female adolescents from Grade 11 from Rural Hunan were included with a prevalence rate of 2.5%. In the present study, prevalence was much higher. This may be attributed to different screening instruments for DEB. The study by Sing Lee and Antoinette M. Lee [6] used EAT-26 as the screening instrument, while we used SCOFF. The sensitivity and the specificity between the two instruments are different and SCOFF has been reported to have a tendency toward overinclusion [34]. It is further comparable to reports from Western countries; in spite of variation in measuring instruments, study methodologies and sample compositions: for instance 29.4% of female adolescents and 14.9% of male adolescents in Germany, and 56% of female adolescents and 28% of male adolescents in US [14, 15]. Given the harmful consequences related to DEB and the high prevalence of DEB, it is vital for public health officials to increase public awareness by introducing relevant and important information about DEB at individual, school and society level. In this way, health organisations can aim at intervening and preventing harmful eating behaviours. So far, there has been no such programs for DEB in the local region.

This study did not show significant gender differences in prevalence of DEB. Most Chinese and Western studies report that prevalence of DEB was significantly higher among female adolescents than among male adolescents [9, 14, 15]. The prevalence of DEB for males is possibly rising and the incidence of DEB for males may have been underrated due to cultural stigma [35]. It may also due to changing lifestyles: increased access to TV, social media, Internet and gaming. It is possible that female adolescents and male adolescents have a similar risk for developing DEB. More attention, therefore, should be also be paid to male adolescents who are at risk of DEB.

The present study found perceived weight as a significant associated factor of DEB. Results are consistent with previous observations on perceived weight status in Chinese adolescents [6] and Western samples [36, 37]. It has been suggested that one’s perceived weight has a greater influence than one’s actual weight when engaging in DEB [38]. In addition, a considerable number of adolescents in this study misperception with regard to weight status, which is consistent with previous Chinese research [39]. Furthermore, separate logistical regression in this study showed that students with the misconception of being overweight were more likely to report DEB. This is in line with previous research [38] and it indicates that one possible avenue for prevention programs may be a focus upon helping students develop accurate weight perception.

Along the same lines, body dissatisfaction was a significant associated factor for DEB. Similar results have been reported in both Chinese and Western research [13, 16]. The thin-ideal image contributes to perfectionism and body dissatisfaction. Male adolescents wish to become more muscular and achieve the male-ideal of V-shaped figure. On the other hand, female adolescents wish to become thinner and achieve the female-ideal of extremely thin. They put much emphasis upon having a perfect body shape, though they have a clinically healthy weight. Perfectionism and body dissatisfaction are both highly correlated with DEB [40]. Body dissatisfaction can increase dieting behaviours [41] and negative affect [16], which can, in turn, lead to poor eating habits and increased eating disturbances.

Negative affect was significantly associated with DEB in this study. The result is in agreement with previous Chinese and Western research [13, 16]. One explanation is that negative urgency (i.e., the tendency to act rashly in response to negative affect) is significantly associated with disordered eating [42]. Another explanation can be derived from escape theory - people tend to escape negative emotions by abnormal eating (overeating) and they believe or have learned that overeating can relieve negative emotions [43, 44]. Moreover, negative affect and disordered eating may be interacted mutually, where the feeling of shame and guilty caused by disordered eating may result in negative self-perceptions among female adolescents [44].

In addition, this study found television access as a significant associated factor of DEB. A small number of adolescents did not have access to TV, possibly related to high academic expectations, family economic situations or personal preference. Media consumption can significantly predict female adolescents’ DEB and male adolescents’ interest in dieting [45]. Such an effect of media can also be found in other non-western studies. For example, research in Fiji [46], Pakistan [47], Japan [48], and Korea [49] have documented significant associations between media exposure and DEB. Becker [50] has also discussed how Western media introduced by TV has caused thin-ideal internalization among Fijian females. Garfinkel and Garner suggest: “The media have capitalized upon and promoted this image (of thinness) and through popular programming have portrayed the successful and beautiful protagonists as thin. Thinness has thus become associated with self-control and success.” (p.145) [51]. Other media variables in this study did not show significant associations with DEB. It is possible that specific media exposure may contribute to thin body and eating disturbances instead of other general media variables.

Although BMI did not contribute to the multiple logistical regression model in this study, BMI was significantly positively associated with DEB from univariate logistical regression analysis. Previous research showed there may exist a U-shaped relation between DEB and BMI [52, 53]. That is, disordered eating behaviours were more problematic at the two extreme ends of BMI (underweight and obesity).

In addition, there is a non-significant trend of increasing prevalence of DEB with age in this study; late adolescents reported highest prevalence of DEB. This may be related to an increased social and emotional stress. Late adolescents, face an exceptional amount of pressure to succeed in college entrance examinations and achieve parental expectations [54] and experience peer competition, copious homework and reduced social support. These stressors may be associated with an increased risk for DEB [55].

This study adds to a growing literature on epidemiology of DEB among adolescents in China. It, therefore, provides current knowledge on the epidemiology of DEB in rural China, which helps to capture more attention to DEB in rural China. Since DEB may develop into more severe forms of DEB or eating disorders without appropriate intervention strategies, it is important to design early interventions against DEB. Secondly, this study included both male adolescents and female adolescents which has provided valuable insights into DEB in both genders. Thirdly, this study found risky behaviours associated with DEB among adolescents, which may contribute to better identification of high-risk groups and may be helpful for early prevention and intervention programs. For instance, adolescents with inaccurate weight perception and body dissatisfaction could be a targeted group for eating problems’ prevention programs. A school-based program could be carried out by helping students reduce weight-related teasing, improve self-esteem and body satisfaction.

This study has some limitations, however. Firstly, the cross-sectional nature of this study could cause difficulty for examining cause-and-effect relationships between DEB and associated factors. Secondly, self-report measures in this study may lead to under- or overestimation of prevalence of DEB and associated factors. For example, self-reported BMI may overestimate measured BMI at the low end of the BMI scale and underestimate measured BMI at the high end [56]. Thirdly, unknown response bias may exist that can reduce the reliability and validity of the study. For example, students may respond untruthfully or give answers that they felt the researchers wanted to hear. Fourthly, the limitation of the SCOFF questionnaire needs to be considered. Since it has shown low sensitivity in a validation population-based study [57]. Fifthly, information on students’ reading level was not available. Since low reading levels may lead to misunderstanding of the questionnaires, the lack of data on reading levels may reduce the reliability of the study. Lastly, this study was school-based and thus access to participants was limited by those who were attending school. No accurate statistical information about school dropout rate was available. According to a literature review in 2015 [58], cumulative dropout rate in rural secondary schools was as high as 63%, while dropout rate in cities was less than 10%. There have been no statistics for students attending vocational and technical schools in China. This sample may be biased towards adolescents with higher education, therefore. The results would be more representative by conducting a population- or community-based study.

## Conclusion

The present study has re-examined DEB among Chinese adolescents in a rural area. It suggests that DEB among rural Chinese adolescents are prevalent. Perceived weight, negative affect, body dissatisfaction and watching TV are significant associated factors for DEB among adolescents. This may provide valuable implications for prevention and intervention strategies in DEB. Furthermore, longitudinal population-based studies are recommended to produce a more comprehensive understanding of causes and risk factors of DEB among Chinese adolescents.

## Additional files

Additional file 1: Table S1. Descriptive statistics of each item of SCOFF. (DOCX 11 kb)

Additional file 2: Table S2. Cross-tabulation of BMI and perceived weight categories among adolescents. (DOCX 11 kb)

Additional file 3: Table S3. Associations between misconception of over−/under-weight and DEB by gender. (DOCX 11 kb)

Additional file 4: Table S4. Multiple logistic regression model showing factors associated with disordered eating behaviours by coefficient and standard errors. (DOCX 13 kb)

## Abbreviations

AUC: Area under the ROC curve
BMI: Body Mass Index
CI: Confidence intervals
DEB: Disordered eating behaviours
df: Degrees of freedom
M: Mean
N: Number
OR: Odds ratio
ROC curve: Receiver operating characteristics curve
sd: Standard deviation
SES: Socioeconomic status
SPSS: Statistical package for the social sciences
VIF: Variance inflation factor
