# Supplementary material for: Eating behaviour disorders among adolescents in a middle school in Dongfanghong, China
Source: J Eat Disord. 2017 Oct 26;5:47. doi: 10.1186/s40337-017-0175-x (PMC5659008; doi:10.1186/s40337-017-0175-x)
Supplement: Supplementary file 1 — Descriptive statistics of each item of SCOFF. (DOCX 11 kb) [file 40337_2017_175_MOESM1_ESM.docx]

**Table S1** Descriptive statistics of each item of SCOFF

| Item of SCOFF | Total  % (N) | Girls  % (N) | Boys  % (N) |
| --- | --- | --- | --- |
| Do you make yourself sick (vomit) because you feel uncomfortably full? (response=yes) | 18.3 (71) | 27.4  (52) | 9.5  (19) |
| Do you worry that you have lost control over how much you eat?  (response=yes) | 23.9 (93) | 27.9 (53) | 20.1 (40) |
| Have you recently lost > 6.35kg in a 3-month period?  (response=yes) | 0.8  (3) | 1.1  (2) | 0.5  (1) |
| Do you believe yourself to be fat when others say you are thin?  (response=yes) | 31.1  (121) | 41.6 (79) | 21.1 (42) |
| Would you say that food dominates your life?  (response=yes) | 27.2  (106) | 14.2 (27) | 39.7 (79) |
