# Supplementary material for: Eating behaviour disorders among adolescents in a middle school in Dongfanghong, China
Source: J Eat Disord. 2017 Oct 26;5:47. doi: 10.1186/s40337-017-0175-x (PMC5659008; doi:10.1186/s40337-017-0175-x)
Supplement: Supplementary file 2 — Cross-tabulation of BMI and perceived weight categories among adolescents. (DOCX 11 kb) [file 40337_2017_175_MOESM2_ESM.docx]

**Table S2** Cross-tabulation of BMI and perceived weight categories among adolescents

|  | Perceived Underweight  % (N) | Perceived  Normal Weight  % (N) | Perceived Overweight  % (N) | Chi-square  Statistic |
| --- | --- | --- | --- | --- |
| Girls-BMI |  |  |  | 55.490*** |
| Underweight | 21.0 (13) | 75.8 (47) | 3.2 (2) |  |
| Normal Weight | 2.2 (2) | 54.8 (51) | 43.0 (40) |  |
| Overweight | 13.8 (4) | 17.2 (5) | 69.0 (20) |  |
| Boys-BMI |  |  |  | 91.209*** |
| Underweight | 38.7 (29) | 50.7 (38) | 10.7 (8) |  |
| Normal Weight | 13.3 (12) | 71.1 (64) | 15.6 (14) |  |
| Overweight | 0.00 (0) | 15.2 (5) | 84.8 (28) |  |

*p < 0.05, **p < 0.01, ****p* < 0.001
