# Supplementary material for: Eating behaviour disorders among adolescents in a middle school in Dongfanghong, China
Source: J Eat Disord. 2017 Oct 26;5:47. doi: 10.1186/s40337-017-0175-x (PMC5659008; doi:10.1186/s40337-017-0175-x)
Supplement: Supplementary file 3 — Associations between misconception of over−/under-weight and DEB by gender. (DOCX 11 kb) [file 40337_2017_175_MOESM3_ESM.docx]

**Table S3** Associations between misconception of over-/under-weight and DEB by gender

|  | OR (95% CI) | *P* |
| --- | --- | --- |
| Girls-Perceived Weight |  |  |
| Underweight | 1 |  |
| Normal Weight | 0.58 (0.20-1.68) | .313 |
| Overweight | 2.20 (0.75-6.42) | .001** |
| Girls-Misconception of Overweight |  |  |
| No | 1 |  |
| Yes | 5.06 (2.43-10.50) | <.001*** |
| Girls-Misconception of Underweight |  |  |
| No | 1 |  |
| Yes | 1.12 (0.20-6.29) | .899 |
| Boys-Perceived Weight |  |  |
| Underweight | 1 |  |
| Normal Weight | 1.44 (0.50-4.17) | .502 |
| Overweight | 11.75 (3.93-35.15) | <.001*** |
| Boys-Misconception of Overweight | 5.95 (3.56-10.24) |  |
| No | 1 |  |
| Yes | 4.91 (1.96-12.33) | .001** |
| Boys-Misconception of Underweight |  |  |
| No | 1 |  |
| Yes | 0.53 (0.11-2.50) | .422 |

Note. OR = odds ratio; CI = confidence interval

**p* < 0.05, ***p* < 0.01, ****p* < 0.001
