# Supplementary material for: Eating behaviour disorders among adolescents in a middle school in Dongfanghong, China
Source: J Eat Disord. 2017 Oct 26;5:47. doi: 10.1186/s40337-017-0175-x (PMC5659008; doi:10.1186/s40337-017-0175-x)
Supplement: Supplementary file 4 — Multiple logistic regression model showing factors associated with disordered eating behaviours by coefficient and standard errors. (DOCX 13 kb) [file 40337_2017_175_MOESM4_ESM.docx]

**Table S4** Multiple logistic regression model showing factors associated with disordered eating behaviours by coefficient and standard errors

|  | Univariate Logistic Regression Analysis per Independent Variable | | Forward Multivariate Stepwise Logistic Regression Analysis | |
| --- | --- | --- | --- | --- |
|  | B | SE | B | SE |
| BMI |  |  |  |  |
| Underweight | - | - | - | - |
| Normal Weight | 0.67 | 0.27 | - | - |
| Overweight | 1.19 | 0.32 | - | - |
| Perceived Weight |  |  |  |  |
| Underweight | - | - | - | - |
| Normal Weight | 0.13 | 0.38 | -0.24 | 0.44 |
| Overweight | 1.69 | 0.38 | 1.02 | 0.50 |
| Weight Misconception |  |  |  |  |
| Correct Weight Conception | - | - | - | - |
| Misconception of Underweight | -0.28 | 0.59 | - | - |
| Misconception of Normal Weight | -0.97 | 0.35 | - | - |
| Misconception of Overweight | 1.35 | 0.30 | - | - |
| Pressure from family | 0.78 | 0.11 | - | - |
| Pressure from friends | 0.91 | 0.13 | - | - |
| Pressure from dating | 0.62 | 0.12 | - | - |
| Pressure from media | 0.64 | 0.11 | - | - |
| Watch TV |  |  |  |  |
| No | - | - | - | - |
| Yes | 0.59 | 0.33 | 0.88 | 0.39 |
| Watch DVD |  |  |  |  |
| No | - | - | - | - |
| Yes | 0.52 | 0.27 | - | - |
| Chat Online |  |  |  |  |
| No | - | - | - | - |
| Yes | 0.86 | 0.34 | - | - |
| Play Online |  |  |  |  |
| No | - | - | - | - |
| Yes | 0.29 | 0.24 | - | - |
| Body Satisfaction | -0.08 | 0.02 | -0.04 | 0.02 |
| Positive Affect | -0.04 | 0.02 | - | - |
| Negative Affect | 0.1 | 0.02 | 0.07 | 0.02 |

Note. B = coefficient ; SE = standard errors

Pressure = perceived sociocultural pressure to thin body and weight loss
